# Sending-or-Not-Sending Twin-Field Quantum Key Distribution with a Passive Decoy-State Method

**DOI:** 10.3390/e24050662

**Published:** 2022-05-08

**Authors:** Ke Xue, Zhigang Shen, Shengmei Zhao, Qianping Mao

**Affiliations:** 1 Institute of Signal Processing Transmission, Nanjing University of Posts and Telecommunications (NUPT), Nanjing 210003, China; 1019010506@njupt.edu.cn (K.X.); shenzg@126.com (Z.S.); 2 Key Lab of Broadband Wireless Communication and Sensor Network Technology, Ministry of Education, Nanjing 210003, China; 3 College of Computer Science and Technology, Nanjing Tech University, Nanjing 211816, China

**Keywords:** sending-or-not-sending, passive decoy-state, quantum key distribution

## Abstract

Twin-field quantum key distribution (TF-QKD) has attracted considerable attention because it can exceed the basic rate-distance limit without quantum repeaters. Its variant protocol, sending or not-sending quantum key distribution (SNS-QKD), not only fixes the security vulnerability of TF-QKD, but also can tolerate large misalignment errors. However, the current SNS-QKD protocol is based on the active decoy-state method, which may lead to side channel information leakage when multiple light intensities are modulated in practice. In this work, we propose a passive decoy-state SNS-QKD protocol to further enhance the security of SNS-QKD. Numerical simulation results show that the protocol not only improves the security in source, but also retains the advantages of tolerating large misalignment errors. Therefore, it may provide further guidance for the practical application of SNS-QKD.

## 1. Introduction

Quantum key distribution (QKD) allows legitimate communicators to share a common key based on the laws of quantum physics [1,2,3]. Thus far, QKD has been developed for nearly 40 years, and great progress has been made both theoretically and experimentally. The emergence of excellent protocols, such as the BB84 protocol [4], Measurement-Device-Independent QKD (MDI-QKD) protocol [5,6,7,8,9] and Round-Robin Differential-Phase-Shift QKD (RRDPS-QKD) protocol [10,11,12], has promoted the progress of QKD. Unfortunately, the above protocols have not broken through the basic rate-distance limit of repeaterless QKD, which is called the PLOB bound [13].

In 2018, the revolutionary twin-field quantum key distribution (TF-QKD) protocol proposed by Lucanarini et al. [14] was able to effectively overcome the PLOB bound, and its experimental secure transmission distance even exceeded 500km. Then, many variant QKD protocols and further studies [15,16,17,18,19,20,21,22] followed to develop the performance of TF-QKD. As the protocol with the longest transmission distance in current QKD experiments, sending-or-not-sending TF-QKD (SNS-QKD) [15,23,24,25,26,27,28,29,30] not only fixes the security vulnerability of TF-QKD, but also tolerates large misalignment errors. Since the single-photon interference is not needed in the signal window [15], SNS-QKD is more practical than other TF-QKD variant protocols.

The QKD systems always adopt the decoy-state method [31,32,33,34,35] to tackle photon-number-splitting (PNS) attacks [36,37], so as to guarantee the security of the light source. Usually, different intensities are actively modulated by the acousto- or electro-optic modulators on the light sources in experiments. Although active modulation is sufficient to achieve decoy-state SNS-QKD, passive modulation of the pulse is still necessary in some cases—for instance, when the intensity modulator is not properly designed so that some physical parameters of the emitted pulses depend on the particular setting [38]. Thus, the active modulation of the pulse intensity at this time will cause severe security problems [38]. Thus far, passive decoy-state methods have been proposed [39,40,41,42,43] and verified [7,44] to be able to reduce the information leakage. However, SNS-QKD using the passive decoy-state method has not been proposed.

Here, in this work, we propose a passive decoy-state SNS-QKD scheme to further improve the security of SNS-QKD in a light source. The scheme uses the heralded single-photon source (HSPS) [45] as a signal source, while the weak coherent state source (WCSs) is still used as a decoy source. The authorized user Alice (or Bob) passively selects whether it is a signal window or a decoy window according to the local detection events occurring at her (his) side. In addition, we compare the performance of the passive scheme with the original SNS-QKD under different conditions, and it is indicated that the proposed protocol retains all the advantages of the original SNS-QKD.

The paper is arranged as follows. In Section 2, we describe the content of our protocol and its settings. We analyze the security of the passive decoy-state method SNS protocol in Section 3 and give the method of calculating the key rate in Section 4. In Section 5, we present the numerical simulation and give an analysis of the these results. Finally, the conclusion is given in Section 6.

## 2. Passive Decoy-State SNS-QKD Protocol

Before introducing our protocol, some assumptions are clarified. First of all, Alice, Bob and Charlie are completely isolated. Secondly, the path of local detection is much shorter than mode S and mode D.

**Figure 1:**
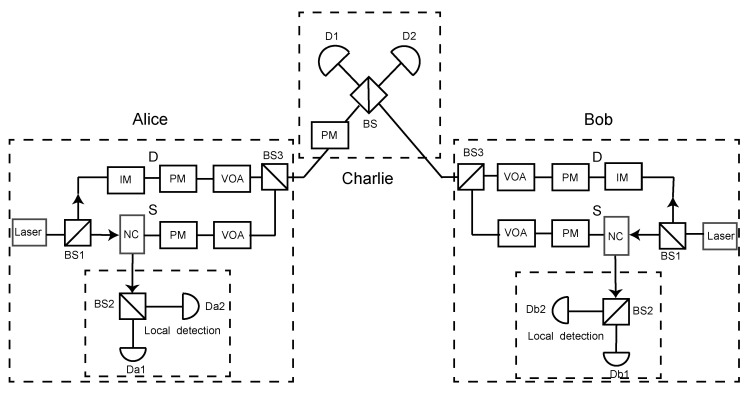
The settings of the passive decoy-state SNS-QKD protcol. BS, beam splitter; IM, intensity modulator; PM, phase modulator; VOA, variable optical attenuator; NC, nonlinear crystal; Local detection, a beam splitter and two local single-photon detectors (Da1 and Da2, Db1 and Db2); D1 and D2, single-photon detectors at Charlie side.

The schematic diagram of passive decoy-state SNS-QKD is shown in Figure 1. Firstly, the pulses are split into two modes (mode D and mode S) by BS1. The mode D is used to send decoy-state pulses, and mode S is used to send signal-state pulses. Next, Alice and Bob passively select one of the modes according to local detection. If Alice (Bob) selects mode D (mode S), she (he) will use the VOA to attenuate the mode S (mode D) pulses. After the pulses are interfered by BS, Charlie announces the results of the successful events. Then, Alice and Bob extract the sifted keys according to the published measurement results. Finally, Alice and Bob can share a secure key after performing error correction and private amplification. The detailed steps of our passive decoy-state SNS-QKD scheme can be described as follows.

Step 1: First of all, the pulses are split into two modes (mode D and mode S) by BS1. The mode D adopts the weak coherent state sources (WCSs) while the mode S adopts the heralded single-photon source (HSPS). The pulses of mode D are modulated by an intensity modulator (IM) and encoded by a phase modulator (PM). The pulses of mode S are further separated into two parts through the parametric down-conversion (PDC) process of the NC. One part (local detection) consists of a beam splitter and two local detectors, and the pulses of another part are then encoded by a phase modulator (PM).

Step 2: Alice (Bob) passively selects whether it is a signal window or a decoy window based on the local detector events. When it is a decoy window, Alice (Bob) randomly chooses one from a few decoy states $|\mu_{m}e^{i\theta_{a}}\rangle$ ($|\mu_{m}e^{i\theta_{b}}\rangle$) ($\mu_{m}\in \mu ,v,0$), which are WCSs. When it is a signal window, Alice (Bob) normally decides to send a signal pulse (HSPS) with a random phase shift $\theta_{a}$ ($\theta_{b}$) by probability ϵ, and she (he) decides to attenuate the pulse by probability 1−ϵ after the detector click.

Note that, as shown in Table 1, the local detection events can be divided into four types, denoted as $E_{i}$ (*i* = 1, 2, 3, 4), corresponding to (1) no response, (2) only Da1 (Db1) response, (3) only Da2 (Db2) response, (4) both responses. Apparently, Alice (Bob) can use these four detection events to passively select whether it is a decoy window or a signal window. After Alice and Bob select the corresponding window, they will use the VOA to attenuate the pulses of the other window. For example, if Alice (Bob) chooses the signal window, she (he) can attenuate the decoy window pulses with a VOA. Moreover, the operation of ‘not sending’ a pulse in the original SNS-QKD [15] is no longer applicable in our protocol because, if no pulse is sent, there will be no local detection event response. Hence, in order to maintain the security equivalence with the original SNS-QKD, we use VOA to attenuate the pulse to zero output to represent the ‘not sending’ operation so as to maintain the completeness of the protocol.

Step 3: Charlie measures the incoming pulses with a BS and announces the measurement results.

Step 4: After the interference by BS, Alice and Bob announce the local detection events and the extra phase of the decoy window.

Note that successful events are defined as the following two situations: (a) both Alice and Bob select the corresponding signal window, and only one detector clicks on Charlie’s side; (b) when Charlie announces that only one detector clicks, Alice and Bob both select the corresponding decoy window, and phases $\theta_{a}$, $\theta_{b}$ satisfy one of the following two equations:

(1) $$\left|\theta_{a}-\theta_{b}\right|\leq \frac{2\pi }{M},\left|\theta_{a}-\theta_{b}-\pi \right|\leq \frac{2\pi }{M},$$

where *M* refers to the total number of phase slices pre-chosen by Alice and Bob.

Step 5: Alice and Bob take some post-processing measures such as error correction and privacy amplification to extract the secure key.

## 3. The Security Analysis

It is known that our protocol maintains most parts of the original SNS protocol except that the active decoy-state method is replaced by the passive-decoy state method. Therefore, we only need to analyze the security problems that are caused by this difference from the original SNS-QKD. Here, we discuss them individually as follows.

(i) The HSPS. In order to implement the passive decoy-state method, we use the HSPS to replace the WCSs of the original SNS in the signal window. Compared with WCPs, the HSPS has a larger single-photon component and smaller vacuum component, which has better performance in the QKD protocol. Many experiments have developed their applications [46,47] with HSPS. The SNS-QKD protocol with the HSPS was further discussed in Ref. [30]. Therefore, the replacement of HSPS will not cause security vulnerabilities of our protocol.

(ii) The passive decoy-state method. Many successful implementations of the passive decoy-state technique in QKD experiments show that it is practical and feasible for the passive decoy-state method [7,44]. Compared with the active decoy-state method, the passive decoy-state method selects the signal state and the decoy state according to the local detection events. This approach not only does not have security vulnerabilities but also improves the security of the protocol. Firstly, the passive decoy-state method can also resist PNS attacks as the eavesdropper cannot distinguish whether the pulse is in the decoy state or signal state. Secondly, it can avoid the security vulnerabilities caused by actively modulating the intensity of the source. For example, Jiang et al. proposed an attack called wavelength-selected photon-number-splitting (WSPNS) in 2012. This attack uses the frequency factor introduced by intensity modulation to distinguish the signal state and decoy state. However, the proposed scheme uses local detection events to distinguish the signal states and decoy states. Therefore, we can make the signal state intensity (laser intensity) the same as one of the decoy state. When both the signal-state intensity and the decoy-state intensity are same (for instance, both intensities are μ), intensity modulation is no longer required. For the vacuum decoy state, the frequency cannot be introduced because there is no pulse. Since there are two decoy states that cannot be distinguished, the WSPNS attack will become ineffective.

(iii) Attenuation of the pulses. In our protocol, the choice of signal state and decoy state needs to be determined according to the response of the local detector. Therefore, we use the VOA to attenuate the pulses instead of not sending pulses. Although the imperfections of the VOA device will decrease the key generation rate of our protocol, it does not leak any information. Thus, attenuation of the pulses has no impact on the security. Additionally, one can reduce the key rate decrease in the post-processing stage.

## 4. The Key Rate

In this part, we will discuss the key rate of the passive decoy-state method SNS-QKD.

### 4.1. The Probability Distribution

In this protocol, we need to deduce the corresponding probability distribution of $E_{1}$ event to analyze the key rate. Following the previous works on QKD with the passive decoy-state method [41,42], we give a brief overview of the derivation process as follows.

Taking the Alice side as an example, suppose that $d_{1}$ and $d_{2}$ are the dark counts of the two local detectors. If the photon number state projected to the Da1 and Da2 detectors is $|s_{1}\rangle |s_{2}\rangle$, the projecting probability $P_{E_{i}|s_{1}s_{2}}$ corresponding to event $E_{i}$ (*i* = 1, 2, 3, 4) can be obtained as shown in Table 2.

For any *n*-photon state, the projecting probability $P_{s_{1}s_{2}|n}$ projecting into state $|s_{1}\rangle |s_{2}\rangle$ can be written as

(2) $$\begin{array}{cc} P_{s_{1}s_{2}|n} & =\sum_{k=0}^{n}\sum_{s_{2}=0}^{n-k}\sum_{s_{1}=0}^{k}C_{n}^{k}t^{k}{\left(1-t\right)}^{n-k}C_{k}^{s_{1}}\eta_{1}^{s_{1}}{\left(1-\eta_{1}\right)}^{k-s_{1}}C_{n-k}^{s_{2}}\eta_{2}^{s_{2}}{\left(1-\eta_{2}\right)}^{n-k-s_{2}}\\ \; & =\sum_{k=0}^{n}\sum_{s_{2}=0}^{n-k}\sum_{s_{1}=0}^{k}\frac{n!t^{k}{\left(1-t\right)}^{n-k}\eta_{1}^{s_{1}}\eta_{2}^{s_{2}}{\left(1-\eta_{1}\right)}^{k-s_{1}}{\left(1-\eta_{2}\right)}^{n-k-s_{2}}}{s_{1}!s_{2}!\left(k-s_{1}\right)!\left(n-k-s_{2}\right)!}, \end{array}$$

where *t* represents the transmittance of BS, and $\eta_{1}$ and $\eta_{2}$ represent the detection efficiency of detector Da1 and detector Da2, respectively.

Therefore, for any *n*-photon state, the probability of obtaining $E_{i}$ event can be written as

(3) $$P_{E_{i}|n}=\sum_{s_{1}s_{2}}^{}P_{E_{i}|s_{1}s_{2}}P_{s_{1}s_{2}|n}.$$

Then, we can obtain the probability distribution of detection events $P_{n}^{E_{1}}$ as

(4) $$P_{n}^{E_{i}}=P_{n}\sum_{s_{1}s_{2}}^{}P_{E_{i}|s_{1}s_{2}}P_{s_{1}s_{2}|n}$$

where $P_{n}$ is the photon-number distribution of the PDC process and it can be either a thermal or Poisson distribution, as introduced in [48,49].

### 4.2. The Parameter Estimation

As shown in Table 3, we take $E_{1}$ as the signal window event and other events as the decoy window events. According to Ref. [15], we can obtain the key rate of our protocol as

(5) $$R=2\epsilon \left(1-\epsilon \right)P_{1}^{E_{1}E_{1}}Y_{1}\left[1-H_{2}\left(e_{1}\right)\right]-Q_{E_{1}E_{1}}fH_{2}\left(E_{E_{1}E_{1}}\right),$$

where $P_{1}^{E_{1}E_{1}}$ is the probability distribution of single-photon states in the signal window, i.e., $P_{n}^{E_{1}E_{1}}=\sum_{k=0}^{n}P_{k}^{E_{1}}P_{n-k}^{E_{1}}$ [27]. *f* is the error correction inefficiency; $H_{2}\left(a\right)=-x\log_{2}a-\left(1-a\right)\log_{2}\left(1-a\right)$; $Y_{1}$ and $e_{1}$ are the yield and error rate of single-photon states. $Q_{E_{1}E_{1}}$ and $E_{E_{1}E_{1}}$ are the total gain and error rate of signal states in the signal window, respectively.

In the decoy window, we still use the WCPs and two weaks + vacuum decoy-state method as the original SNS protocol. According to the previous decoy-state method [7,15,24,27,32,33], we can obtain the lower bound of $Y_{1}$ and the upper bound of $e_{1}$ as follows:

(6) $$\begin{array}{c} Y_{1}\geq \frac{P_{2}^{\mu }\left(Q_{v}-P_{0}^{v}Y_{0}\right)-P_{2}^{v}\left(Q_{\mu }-P_{0}^{\mu }Y_{0}\right)}{P_{2}^{\mu }P_{1}^{v}-P_{2}^{v}P_{1}^{\mu }}, \end{array}$$

(7) $$e_{1}\leq \frac{Q_{v}E_{v}-P_{0}^{v}Y_{0}e_{0}}{P_{0}^{v}Y_{1}},$$

where the subscript 0 indicates that Alice and Bob prepare a vacuum state. $P_{n}^{\mu }$ (*n* = 0, 1, 2) is the probability distribution of intensity μ, and the total photon number is *n*. $Q_{\mu }$ ($Q_{v}$) and $E_{\mu }$ ($E_{v}$) are the gain and quantum bit error rate (QBER) of intensity μ (*v*).

## 5. Numerical Simulations

In this part, we present some results of the numerical simulation. Here, we focus on the symmetric case, which means that the device parameters at the Alice side and Bob side are identical. To simplify the calculation, we also let $d_{1}=d_{2}=d_{L}$ and $\eta_{1}=\eta_{2}=\eta_{L}$. According to Equation (4), we can obtain the simplified probability distribution $P_{n}^{E_{1}}$ for the Alice side and Bob side as

(8) $$P_{n}^{E_{1}}=C_{\mu }{\left(1-d_{L}\right)}^{2}{\left(1-\eta_{L}\right)}^{n}P_{n},$$

where $P_{n}$ is a Poissonian distribution, i.e., $P_{n}=\frac{u^{n}}{n!}e^{-u}$. Here, $C_{\mu }$ is the normalization factor, which is $C_{\mu }^{-1}=\sum_{n=0}^{\infty }P_{n}^{E_{1}}$.

Next, we derive the values that should be observed in the experiment. According to Refs. [24,26], the corresponding gains and the QBERs in the signal window are given by

(9) $$\begin{array}{cc} Q_{E_{1}E_{1}} & =\;{\left(1-\epsilon \right)}^{2}Y_{0}+4\epsilon \left(1-\epsilon \right)\left(1-d_{c}\right)\sum_{n}^{}P_{n}^{E_{1}}{\left(1-\eta \right)}^{n}\sum_{n}^{}P_{n}^{E_{1}}\left(1-\left(1-d_{c}\right){\left(1-\eta \right)}^{n}\right)\\ & +\;2\epsilon^{2}\left(1-d_{c}\right)\sum_{n}^{}P_{n}^{E_{1}E_{1}}{\left(1-\eta \right)}^{n}\sum_{n}^{}P_{n}^{E_{1}E_{1}}\left(1-\left(1-d_{c}\right){\left(1-\eta \right)}^{n}\right), \end{array}$$

(10) $$\begin{array}{c} E_{E_{1}E_{1}}Q_{E_{1}E_{1}}={\left(1-\epsilon \right)}^{2}Y_{0}+2\epsilon^{2}\left(1-d_{c}\right)\sum_{n}^{}P_{n}^{E_{1}E_{1}}{\left(1-\eta \right)}^{n}\sum_{n}^{}P_{n}^{E_{1}E_{s}}\left(1-\left(1-d_{c}\right){\left(1-\eta \right)}^{n}\right), \end{array}$$

where $Y_{0}$ is the yield of the vacuum pulse; $d_{c}$ is the dark count rate of detectors at the Charlie side; η is the total system efficiency, which is $\eta =\eta_{d}\eta_{c}$; $\eta_{d}$ is the detection efficiency in Charlie’s part; $\eta_{c}$ is the transmittance of the quantum channel, $\eta_{c}=10^{-\frac{\frac{\alpha l}{2}}{10}}$, α is the fiber loss coefficient and *l* is the distance between Alice and Bob. The device parameters used in numerical simulations are listed in Table 4.

**Figure 2:**
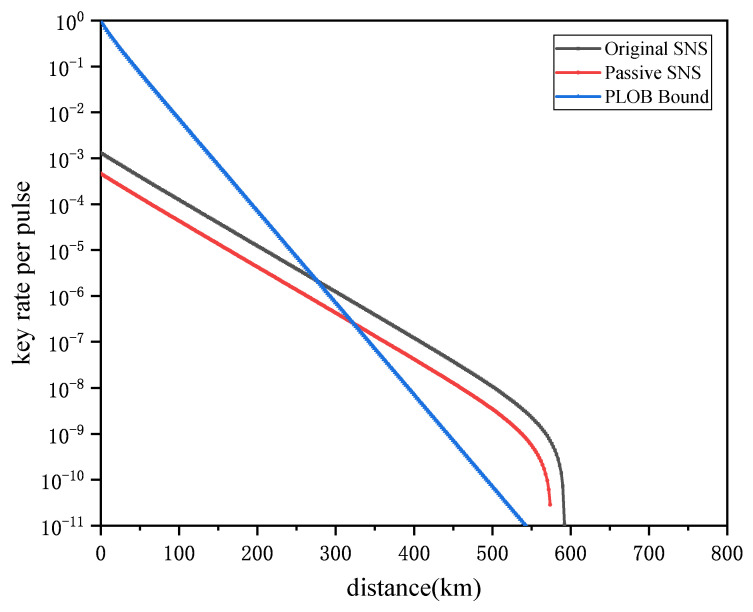
The performance of the passive decoy-state SNS-QKD compared to original SNS protocol.

Figure 2 shows the comparison of the key rates between the passive decoy-state SNS-QKD and the original SNS-QKD. Note that the performance of our scheme is also related to the quality of local detectors, e.g., the dark count rate and the detection efficiency. The simulation results indicate that, with the passive decoy-state scheme, our protocol can have a performance that is close to that of the original SNS protocol. Moreover, they all exceed the PLOB limit when l≥300 km.

**Figure 3:**
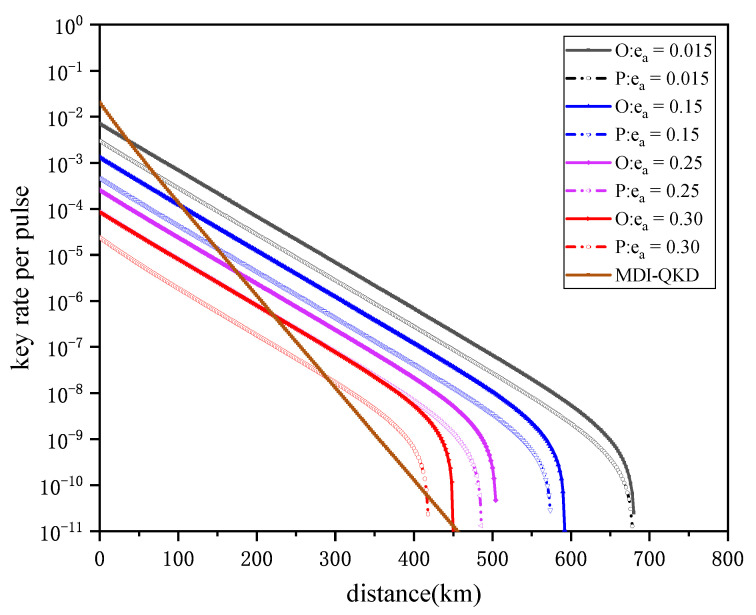
The performance of both original SNS protocol and passive decoy-state SNS-QKD is simulated under misalignment errors $e_{a}$. P: Passive decoy-state SNS-QKD. O: Original SNS-QKD. MDI-QKD: MDI-QKD with active decoy-state method in coherent states.

Figure 3 shows the performance of both the original SNS protocol and passive decoy-state SNS-QKD simulated under misalignment errors $e_{a}$. The misalignment error rates are set as 0.015, 0.15 and 0.30, respectively. Moreover, we also add an MDI-QKD curve for comparison. For the key rate of MDI-QKD, the misalignment error rate of X-basis is set to 0.015 and Z-basis is set to 0. The numerical results show that passive decoy-state SNS-QKD still performs well under different misalignment errors, as its performance remains close to the original SNS protocol. In addition, compared with the MDI-QKD, our protocol has better key rate performance, even if the misalignment error is as high as 0.25. In other words, the proposed protocol can still tolerate quite high misalignment errors.

**Figure 4:**
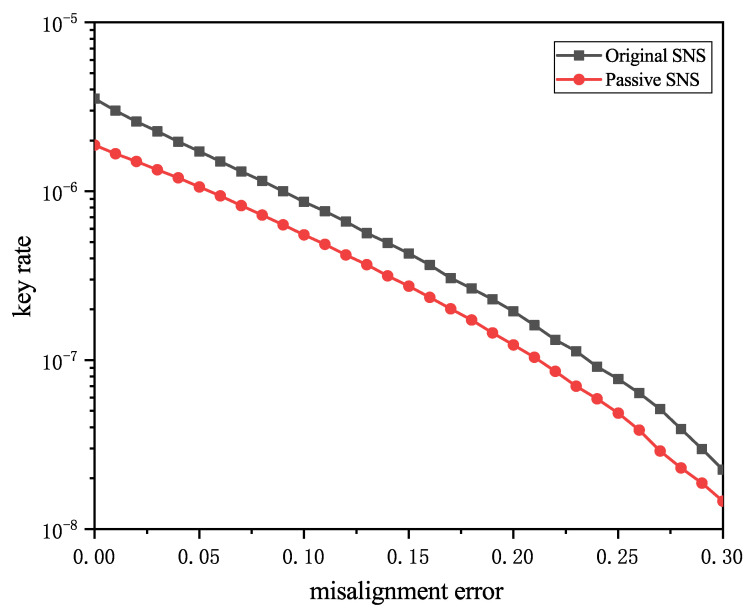
Key rates as a function of the misalignment error when the distance between Alice and Bob is 300 km.

Figure 4 shows the comparison of the tolerance of passive decoy SNS-QKD and original SNS-QKD to misalignment errors $e_{a}$. The simulation distance is set to 300 km. Obviously, the SNS-QKD with the passive decoy-state scheme is very close to the original SNS-QKD protocol. Even if the misalignment error reaches 0.30, the performance of the two protocols is still good. This shows that the proposed protocol still retains the advantages of the original SNS-QKD protocol, indicating that the SNS protocol has a broader prospect in implementing long-distance QKD.

## 6. Conclusions

In summary, we have proposed the passive decoy-state SNS-QKD protocol to enhance the security of the source of the SNS-QKD protocol. We have presented the framework of the passive decoy-state SNS-QKD protocol and have analyzed the security of the proposed protocol. The numerical simulation results have demonstrated that the key rates of our proposed protocol are close to the original SNS-QKD, which uses the active decoy-state method. Moreover, our protocol can tolerate larger misalignment errors in QKD systems, indicating that the SNS protocol can still have a long transmission distance with a passive decoy-state in real-life QKD systems. Therefore, our protocol represents a further step toward the application of the SNS-QKD.

## Figures and Tables

**Table 1:** Definition of various detection events. Taking Alice side as an example, 0 indicates that the detector has not clicked, and 1 indicates that the detector has clicked.

Events	Da1	Da2
$E_{1}$	0	0
$E_{2}$	1	0
$E_{3}$	0	1
$E_{4}$	1	1

**Table 2:** Probability of the $E_{i}$ (*i* = 1, 2, 3, 4) event occurring.

Case	$P_{E_{1}|s_{1}s_{2}}$	$P_{E_{2}|s_{1}s_{2}}$	$P_{E_{3}|s_{1}s_{2}}$	$P_{E_{4}|s_{1}s_{2}}$
$s_{1}=0,s_{2}=0$	$\left(1-d_{1}\right)\left(1-d_{2}\right)$	$d_{1}\left(1-d_{2}\right)$	$\left(1-d_{1}\right)d_{2}$	$d_{1}d_{2}$
$s_{1}\neq 0,s_{2}=0$	0	$\left(1-d_{2}\right)$	0	$d_{2}$
$s_{1}=0,s_{2}\neq 0$	0	0	$\left(1-d_{1}\right)$	$d_{1}$
$s_{1}\neq 0,s_{2}\neq 0$	0	0	0	1

**Table 3:** Definition of signal window and decoy window based on the detection events.

Sender	Signal Window	Decoy Windows
Alice	$E_{1}$	other events
Bob	$E_{1}$	other events

**Table 4:** Device parameters used in numerical simulations.

α	$e_{0}$	$\eta_{d}$	$\eta_{L}$	$d_{c}$	$d_{L}$	*f*
0.2	0.5	0.5	0.5	$10^{-10}$	$10^{-10}$	1.10

## Data Availability

Not applicable.
